# Amyand's Hernia in a 6-Week-Old Infant: A Delayed Diagnosis

**DOI:** 10.1155/2013/758171

**Published:** 2013-02-07

**Authors:** Cláudia Piedade, Júlio Reis Alves

**Affiliations:** Department of Pediatric Surgery, Pediatric Hospital, Centro Hospitalar e Universitário de Coimbra, Avenida Afonso Romão, 3000-602 Coimbra, Portugal

## Abstract

In Amyand's hernia, the hernia sac contains the appendix. This can be normal and accidentally found during herniotomy or inflamed and present as acute scrotum, although the latter is less frequent. We present a case of a male infant with scrotal abscess resulting from Amyand's hernia, with appendicitis and periappendicular abscess.

## 1. Introduction

Inguinal hernia is the most common condition encountered in infants, requiring early and special attention, because of the risk of incarceration and/or strangulation, with consequent significant morbidity. Amyand's hernia is an inguinal hernia in which the hernia sac contains the appendix, and which is extremely rare in children, especially in infants. This phenomenon was described in 1735, when Claudius Amyand performed a successful appendectomy on an 11-year-old boy who presented with an appendix in a hernia sac. So, in his honour, his name was given to this type of hernia. 

Diagnosis can be difficult due to its rarity, and the presentation is similar to that of any inguinal hernia, with tenderness, erythema, and inability to reduce contents, if incarcerated. When appendicitis occurs, it can too mimic testicular inflammation or torsion, and so preoperative diagnosis is very rare. 

Hernia repair and appendectomy are the treatment of choice. Antibiotherapy is associated, in order to prevent complications, such as intra-abdominal abscess.

## 2. Case Report

A 6-week-old male, born at 36-week gestation, was presented to our emergency department with a diagnosis of “right strangulated and perforated inguinal-scrotal hernia.”

**Figure 1:**
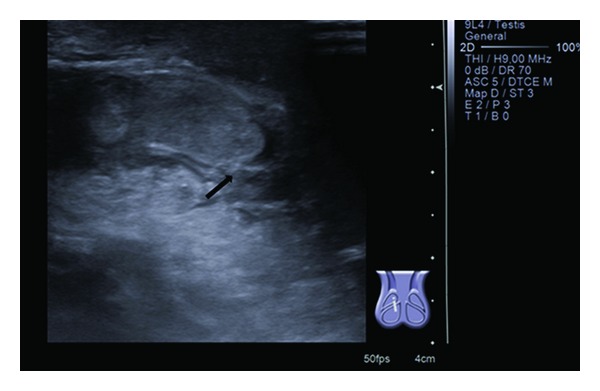
Scrotal ultrasound: scrotal inflammation—arrow.

About 17 days before, he had already presented to our hospital with a right inguinal swelling compatible with incarcerated inguinal hernia. The reduction was performed successfully. Three days after this episode, because of persistent oedema and inflammation of the scrotal soft tissue, his parents went to a private hospital. Ultrasound showed epididymitis and hypervascularization of the inguinal cord. He was therefore medicated with amoxicillin (60 mg/kg/day) and completed 15 days of antibiotherapy with apparent improvement. Two days later, the child started a fever, vomiting, and aggravated inguinal swelling. Ultrasound done at the private hospital showed a right inguinal hernia, testicle and epididymis with inflammation, and heterogeneous fluid in the hernia sac, findings which are compatible with incarcerated and perforated inguinal hernia or possibly an abscess (Figure 1). The child was therefore referred to our hospital. 

Upon observation, the right hemiscrotum was swollen, painful, and hard with the central area compatible with abscess. We decided to subject the patient to surgery with a preoperative diagnosis of “strangulated inguinal hernia with bowel perforation or orchiepididymitis complicated with abscess.”

**Figure 2:**
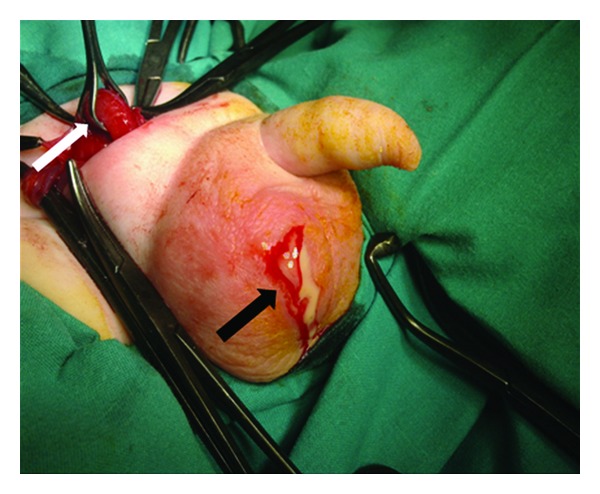
Purulent fluid of hemiscrotum—black arrow and appendix in hernia sac—white arrow.

**Figure 3:**
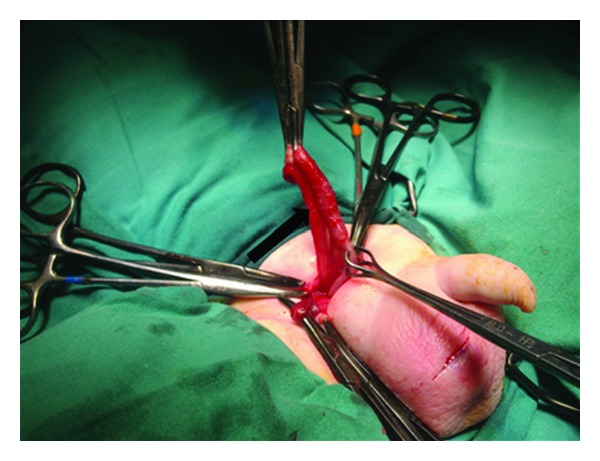
The contents of hernia sac: appendix—arrow.

Surgical exploration was performed initially through a right scrotal incision and revealed a purulent fluid in the right hemiscrotum (Figure 2) and a gangrenous, perforated appendix, especially on the distal third. The purulent fluid was aspirated and the scrotum explored. The testicle showed no evidence of ischemia or significant inflammation. The inguinal canal was opened through a transverse lower abdominal incision, and it was confirmed that the hernia sac contained only the appendix (Figure 3). 

Appendectomy, taking every precaution to avoid the risk of peritoneal contamination, high ligation of the hernia sac, and scrotal drainage were performed. Intravenous antibiotics (gentamicin, ampicillin, and metronidazole) were administered for 4 days. Culture of fluid grew *Staphylococcus aureus *and* Klebsiella pneumonia.*

The patient had an uneventful postoperative course and was discharged on the 4th postoperative day. He continued oral antibiotics (amoxicillin and clavulanic acid, 65 mg/kg/day) during 7 days. On the 15th postoperative day, the patient had no evidence of complications. We maintained followup until 1 year after surgery, and the patient did not have any complications.

## 3. Discussion

The presence of normal appendix in the hernia sac is a rare condition, with an incidence of about 1%. Much rarer is the case of an appendix complicated by acute appendicitis (AA) and periappendicular abscess, with an incidence between 0.08 and 0.13 percent, but an accurate incidence cannot be estimated because few cases have been reported in the literature [1]. 

AA in infants less than 1 year old represents 2% of the total cases of appendicitis. Generally, AA is associated with Hirschsprung's disease, necrotizing enterocolitis, or hernia sac with incarcerated appendix, the latter representing about 1/3 of the cases [2, 3].

Due to the low incidence of appendicitis in these infants and high incidence of incarcerated inguinal hernia, the diagnosis of appendicitis in the hernia sac is delayed, as in this case, and so the risk of perforation is higher. 

In the literature, cases of acute appendicitis in the hernia sac, in children of ages varying between 3 weeks and 18 years, affect the male sex more often, as inguinal hernia. In our centre, this is the only case in the last 10 years. 

The pathophysiology of Amyand's hernia and its relationship with appendicitis are unknown. Some authors consider it as an accidental finding; for others, a decrease in vascularization during incarceration and the manoeuvre to reduce the hernia result in the inflammation of the appendix [4]. In this case, clinical evolution shows that probably in the first time of incarceration the appendix has not been reduced and caused oedema and local inflammation, with compromised vascularization of the appendix, inflammation, and perforation. Moreover, abscess only in the hernia sac reinforces this aspect. 

As clinical presentation is variable, depending on inflammation with or without perforation, a definite diagnosis is often made during surgery. In fact, it can present as incarcerated hernia, scrotal abscess. So, in infants less than 1 year old, who present with recurrent painful swelling of the inguinal region, we have to suspect this entity. In our case, clinical evolution and the prior suspected diagnosis by ultrasound of orchiepididymitis masked and delayed a definitive diagnosis [5, 6]. 

As in other cases of acute scrotum, treatment is surgical inguinal exploration, followed by hernia repair and appendectomy. If peritoneal contamination is presented, additional laparotomy may be necessary. 

This case highlights the need to consider alternative diagnoses in acute scrotum and supports the practice of surgical exploration appropriate for each case.
